# Functional analyses of glycosylated gag molecules derived from different gammaretroviruses

**DOI:** 10.1186/1742-4690-10-S1-P22

**Published:** 2013-09-19

**Authors:** Emilia Cristiana Cuccurullo, Mauro Pistello, Yasuhiro Takeuchi, Takayuki Miyazawa, Pizzato Massimo

**Affiliations:** 1 Centre of Integrative Biology, University of Trento, Trento, Italy; 2 Department of Translational Research, Retrovirus Center and Virology Section, University of Pisa, Pisa, Italy; 3 MRC/UCL Centre for Medical Molecular Virology and Wohl Virion Centre, Division of Infection & Immunity, UCL Cancer Institute, University College London, London, UK; 4 Department of Cell Biology, Institute for Virus Research, Kyoto University, Kyoto, Japan

## Background

Nef is a regulatory lentiviral protein crucial for sustained virus replication *in vivo* and for disease progression. Among its many activity, Nef downregulates CD4 and MHC-I, activates cellular kinases and enhances virus infectivity. While Nef is uniquely found in the genome of primate lentiviruses, glycosylated gag of MoMLV is an infectivity factor functionally related to Nef, which can rescue the activity of Nef-defective HIV-1. Since a putative ORF-encoding glycosylated gag can be found in the genome of diverse exogenous and endogenous gammaretroviruses, we investigated whether the Nef-like activity on retrovirus infectivity is conserved in different glycosylated gag alleles and whether such activity can be linked to a conserved featured of the aminoacid sequence. Additionally we are searching for Nef-like factors in other lymphotropic retroviruses which display pathogenic features similar to HIV.

## Materials and methods

We have tested the ability of diverse gammaretroviruses to encode glycosylated gag molecules and analyzed their ability to increase retrovirus infectivity. The activity of glycosylated gag was tested both either in the context of proviral expression or as ectopically over-expressed molecules in HIV-1 producing cells. Expression level and intracellular localization were also studied and compared.

## Results

We found that all glycosylated gag molecules tested have a measurable Nef-like activity on the infectivity of HIV-1. Accordingly, despite low aminoacid sequence conservation, all protein sequences are predicted to have a type II transmembrane topology and were found to prominently localize in intracellular perinuclear compartments, similarly to Nef. However, the magnitude of the activity on retrovirus infectivity of molecules derived from different gammaretroviruses is highly variable. Finally, using a minimal active MoMLV glycosylated gag carrying a heterologous membrane anchor, we established that both external and transmembrane domain are dispensable for the Nef-like activity.

## Conclusions

The activity on retrovirus infectivity is a conserved feature of glycosylated gag of gammaretroviruses and depends on the cytoplasmic tail of the protein.

